# Incidence and risk factors for postoperative delirium after head and neck cancer surgery: an updated meta-analysis

**DOI:** 10.1186/s12883-023-03418-w

**Published:** 2023-10-17

**Authors:** Bo Dong, Dongdong Yu, Li Jiang, Meinv Liu, Jianli Li

**Affiliations:** Department of Anesthesiology, Hebei General Hospital, Shijiazhuang city, China

**Keywords:** Risk factors, Incidence, Postoperative delirium, Meta-analysis, Systematic review, Head and neck cancer

## Abstract

**Background:**

Postoperative delirium (POD) is a frequent neurologic dysfunction that often leads to more negative outcomes. Early identification of patients who are vulnerable to POD and early implementation of appropriate management strategies could decrease its occurrence and improve patient prognosis. Therefore, this meta-analysis comprehensively and quantitatively summarized the prevalence and related predictive factors of POD in head and neck cancer surgical patients.

**Methods:**

PubMed, Embase, and Cochrane Library were searched for observational studies that reported the prevalence and risk factors for POD after head and neck cancer surgery and were published from their inception until December 31, 2022. Two reviewers independently selected qualified articles and extracted data. The qualities of related papers were assessed using the Newcastle-Ottawa scale (NOS). RevMan 5.3 and Stata 15.0 were applied to analysis the data and conduct the meta-analysis.

**Results:**

Sixteen observational studies with 3289 inpatients who underwent head and neck cancer surgery were included in this review. The occurrence of POD ranged from 4.2 to 36.9%, with a pooled incidence of 20% (95% CI 15–24%, I^2^ = 93.2%). The results of this pooled analysis demonstrated that the statistically significant risk factors for POD were increased age (OR: 1.05, 95% CI: 1.03–1.07, *P* < 0.001), age > 75 years (OR: 6.52, 95% CI: 3.07–13.87, *P* < 0.001), male sex (OR: 2.29, 95% CI: 1.06–4.97, *P* = 0.04), higher American Society of Anesthesiologists grade (OR: 2.19, 95% CI: 1.44–3.33, *P* < 0.001), diabetes mellitus (OR: 2.73, 95% CI: 1.24–6.01, *P* = 0.01), and history of smoking (OR: 2.74, 95% CI: 1.13–6.65, *P* = 0.03).

**Conclusions:**

POD frequently occurs after head and neck cancer surgery. Several independent predictors for POD were identified, which might contribute to identifying patients at high risk for POD and play a prominent role in preventing POD in patients following head and neck cancer surgery.

**Supplementary Information:**

The online version contains supplementary material available at 10.1186/s12883-023-03418-w.

## Introduction

 Head and neck cancer is one of the most frequent malignancies, including cancers of the oral cavity, pharynx, larynx, paranasal sinuses, and nasal cavity [1]. There were more than 878,000 new cases of head and neck cancers in 2020 and approximately 445,000 deaths each year worldwide [2]. Over the past few decades, researchers have made great efforts to explore therapeutic strategies for head and neck cancer, such as radiotherapy, chemotherapy, and immunotherapy; however, surgical resection is still the main treatment method [3]. Unfortunately, owing to the complex nature, multiple comorbidities, highly invasive and extensive surgical procedures, and longer operation time, surgery may inevitably lead to postoperative complications, which not only prolong the hospital stay and decrease the quality of life but also increase the total hospital cost and the risk of mortality [4]. Postoperative delirium (POD), a relatively frequent neuropsychiatric disorder after anesthesia and surgery, is an acute and transient cerebral disorder characterized by disturbance of attention, perception, and consciousness [5]. It was reported that approximately 11.50 to 36.11% of inpatients experienced POD after head and neck cancer surgery, depending on the frequency of assessment, age of the patient, and different types of surgical interventions [6]. POD can lead to unfavourable events such as prolonged hospital stay, increased risk of dementia, mortality, high medical expenses, functional impairment, and other clinical complications [7, 8]. Fortunately, 30–40% of POD can be prevented by early identification and treatment of its related risk factors, although the present pathophysiology of POD remains obscure [9]. Therefore, it is reasonable to believe that early identification of patients at risk for POD and timely implementation of targeted intervention strategies might play critical roles in reducing POD incidence and its related detrimental effects.

Based on different clinical psychomotor behaviours, POD could be further categorized into three subtypes: hyperactive, hypoactive, or mixed. The subtype of POD may be influenced by factors related to specific surgical patient populations. For example, hypoactive-type POD, characterized by lethargy, apathy, and reduced motor activity, occurs more frequently after cardiac and hip fracture surgery [10, 11], while hyperactive-type POD, characterized by agitation, restlessness, and insomnia, is common after head and neck cancer surgery [12]. Recently, several meta-analyses demonstrated that some predisposing risk factors (ageing, low albumin, diabetes, history of delirium, preoperative depression, preoperative functional dependence, mild cognitive impairment, and carotid artery stenosis) and precipitating risk factors (time of mechanical ventilation, surgery delay > 48 h, and intensive care unit stay time) could increase the incidence of POD in patients after cardiac and orthopedic surgery [13, 14]. In regard to patients undergoing head and neck cancer surgery, there may be different risk factors for POD. Risk factors for POD after head and neck cancer surgery were reported in the individual studies, but the results were inconsistent or even conflicting [12, 15]. Additionally, in 2017, Zhu et al. identified several potential risk factors for POD after head and neck cancer surgery using univariate analysis [6]. However, this meta-analysis only included 8 articles, which contributed to the unreliability of results. In addition, the majority of included studies in this review were from Japan in this review, which might reduce the generalizability of the conclusions. Furthermore, this review used univariable analysis to summarize the risk factors for POD rather than multivariate analysis, which led to the results being less mathematically robust. Over the past five years, several studies reporting the risk factors for POD after head and neck cancer surgery have been published, which may offer some new evidence. Consequently, this study was conducted to comprehensively and quantitatively analyze the prevalence and related risk factors for POD in patients who underwent head and neck cancer surgery, and thus providing guidance for clinical prevention decision-making.

## Methods

Our meta-analysis strictly complied with the guidelines of the Preferred Reporting Items for Systematic Reviews and Meta-Analyses (PRISMA) [16].

### Literature search

PubMed, Cochrane Library, and Embase were comprehensively searched for articles published from their inception until December 31, 2022. Based on the combination of medical subject heading terms and text words, a basic search strategy was conducted using the following terms: “delirium”, “postoperative delirium”, “mixed origin delirium”, “head and neck”, “neoplasms”, “cancer”, and “risk factors”, etc. See Additional File 1 for the detailed search strategies.

### Study selection

Inclusion criteria included the following: (1) studies designed as cohort, case-control, or cross-sectional studies; (2) studies including patients undergoing surgery for head and neck cancer; (3) studies reporting the prevalence and risk factors for POD in patients undergoing surgery for head and neck cancer; (4) studies in which POD was diagnosed by some validated methods, such as the Nursing Delirium Screening Scale (Nu-DESC), Intensive Care Delirium Screening Checklist (ICDSC), Confusion Assessment Method (CAM) or Diagnostic and Statistical Manual of Mental Disorders (DSM); (5) studies written in English; and (6) studies with complete data that could be extracted, including ORs of multivariable risk factors with 95%CIs. Exclusion criteria included the following: (1) reviews, letters, abstract-only publications, animal experiments, and case reports; (2) studies with overlapping populations or duplicate publications; (3) studies that did not investigate the predictors for POD by multivariate logistic regression analysis; and (4) articles with insufficient data for statistics.

### Data extraction and quality assessment

Two authors screened the full text of the articles, extracted the data and assessed the quality of the papers separately. The extracted data comprised authors, year of publication, country, study design, sample size, mean age of the patients, diagnostic methods for POD and its incidence, risk factors for POD, and study quality score. Since all the selected studies were observational studies, the quality of the eligible papers was rated using the Newcastle-Ottawa Scale (NOS), which is recognized as a standardized method for the quality assessment of non-randomized studies [17]. The maximum total score for the included studies was 9 points using the NOS which contains eight items. Papers with NOS scores ≥ 7.0 were considered high quality, and NOS scores < 7.0 were regarded as low quality. Any disagreements were eventually resolved through discussion or negotiation.

### Statistical analysis

Stata 15.0 and RevMan 5.3 were used to analyze all data. If multivariable risk factors were reported in more than two studies, we performed a meta-analysis. Pooled ORs with corresponding 95% CIs were applied to assess the relationship between the predictors and POD, and *P* < 0.05 was regarded as statistically significant. I^2^ values and Q-test statistics were applied to detect heterogeneity among articles, where *P* < 0.1 and I^2^ > 50% were deemed to indicate significant heterogeneity. If the articles showed high heterogeneity, a random-effect model analysis was utilized; otherwise, a fixed-effect model was chosen. The final result for each relevant variable was presented as forest plots. When the heterogeneity of the pooled effect was significant (I^2^ > 50%), we further explored the source of heterogeneity using the sensitivity or subgroup analysis. Publication bias with a funnel plot was also conducted.

## Results

### Literature search

**Fig. 1:**
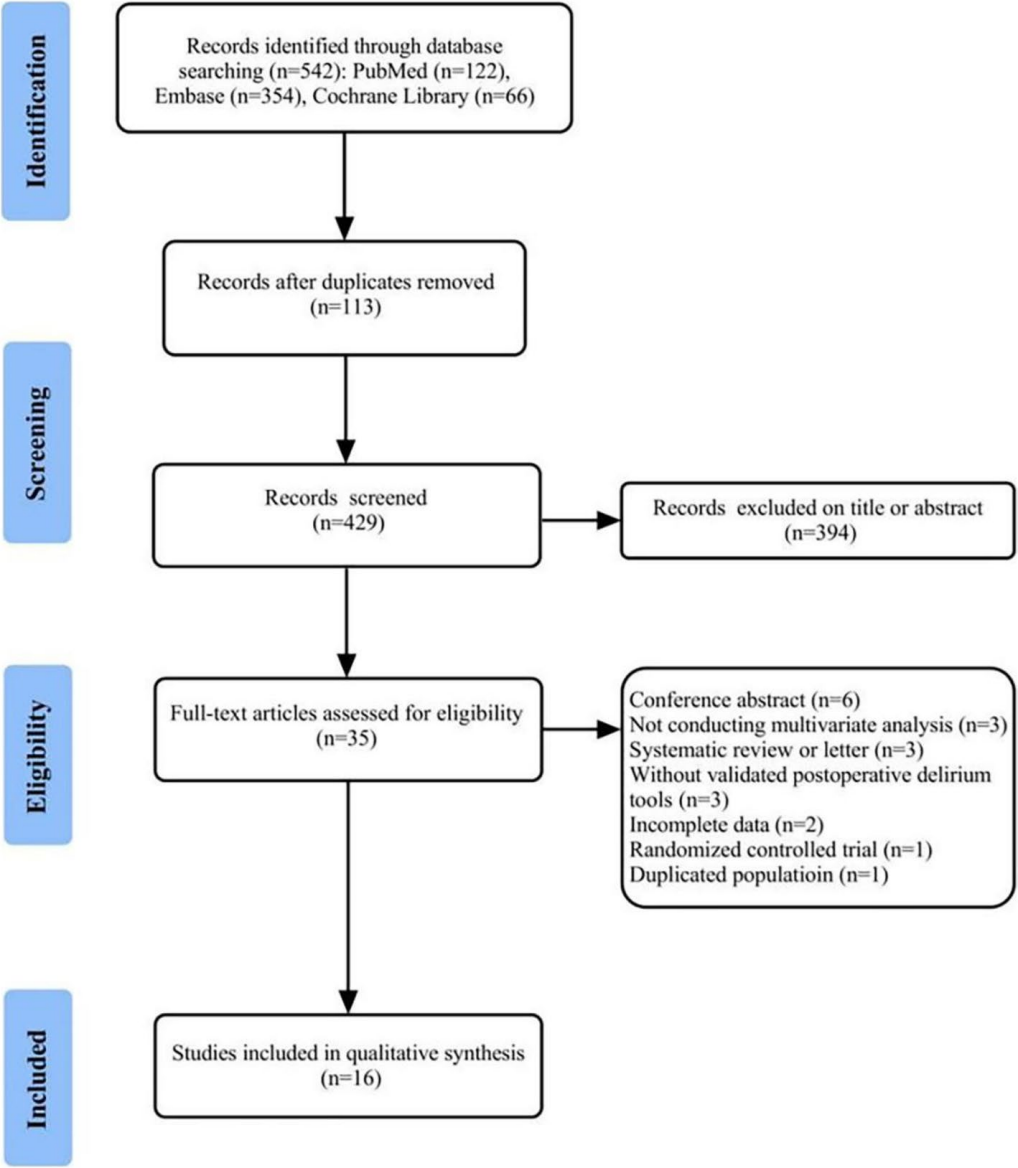
The flow diagram of the search process of the literature and the results of the literature search

The initial literature search retrieved 542 citations from the PubMed (*n* = 122), Embase (*n* = 354), and Cochrane Library (*n* = 66). After the removal of duplicate articles (*n* = 113) by EndNote X9, 429 articles were retained. After preliminary headline and abstract screening, 394 studies were eliminated. The 35 remaining studies consequently underwent full-text review. Subsequently, 19 papers were eliminated for the following reasons: conference abstract (6 studies); not conducting multivariate analysis (3 studies); systematic review or letter (3 studies); without validated POD tools (3 studies); incomplete data (2 studies); randomized controlled trial (1 study);and duplicated population (1 study). Ultimately, 16 articles were eligible for this meta-analysis. The detailed process of the database search is shown in Fig. 1.

### Characteristics of identified studies

In total, 16 observational studies with 3289 patients were published from 2009 to 2022, and the sample size ranged from 69 to 515. Of these 16 observational studies included, 1 was a case-control study, 14 were retrospective cohort studies, and 1 was a prospective cohort study. Of the 16 articles, 12 studies were conducted in Asia (7 in Japan [12, 18–23], 3 in China [24–26], and 2 in South Korea [27, 28]), while 3 studies were performed in the Germany [5, 15, 29] and the remaining 1 study was conducted in the United States [30]. The most common diagnostic method was the DSM-IV in 8 of 16 included studies [12, 18, 19, 21, 22, 24, 27, 30], the DSM-V in 4 studies [5, 15, 20, 29], the CAM in 2 studies [25, 26], and the ICDSC [23] and Nu-DESC [29] in the remaining 2 studies. Table 1 presents the basic information of the selected literature.

**Table 1:** Characteristics of the included studies

Study(year)	Country	Study design	Sample size(n)	Age(years)	POD assessment	POD incidence(%)	Risk factors	Nos score
Shiiba et al. (2009) [18]	Japan	Retrospective	132	63.0 ± 12.6	DSM-IV	18.0	Age, sex	9
Hasegawa et al. (2015) [19]	Japan	Retrospective	188	NR	DSM-IV	15.4	Age, intraoperative hemoglobin, excessive hemorrhage	9
Booka et al. (2016) [20]	Japan	Retrospective	293	61.9 ± 13.6	DSM-V	17.1	Age	9
Choi et al. (2017) [27]	South Korea	Retrospective	341	56 ± 12	DSM-IV	26.0	Age, psychiatric disorder history, marital status, NRS, ASA status, ICU stay period	9
Zhang et al. (2019) [24]	China	Retrospective	287	NR	DSM-IV	4.2	Comorbidity, ASA status	8
Wang et al. (2019) [25]	China	Prospective	323	60.0	CAM	8.7	ASA status, educational level, cancer stage, intraoperative hypotension, intraoperative dexmedetomidine use	8
Ishibashi-Kanno et al. (2020) [12]	Japan	Retrospective	69	62.9 ± 11.9	DSM-IV	33.3	Age, sex, diabetes mellitus, COPD, recent hospitalization, sedation period	9
Densky et al. (2019) [30]	United States	Retrospective	515	60.1 ± 12.8	DSM-IV	10.9	Age, operative time, CCI, sex, tumor N classification, history of smoking,	8
Makiguchi et al. (2020) [21]	Japan	Retrospective	122	60.3 ± 11.2	DSM-IV	36.9	High preoperative albumin, postoperative insomnia, history of smoking, diabetes mellitus	8
Kong et al. (2021) [26]	China	Case-control	98	68	CAM	30.6	Hypertension, irregular medication	7
Takahashi et al. (2021) [22]	Japan	Retrospective	104	63.0	DSM-IV	21.2	Operative time, anesthesia time, blood loss, method of reconstruction, postoperative ambulation, red blood cell count, hemoglobin, hematocrit	8
Kinoshita et al. (2021) [23]	Japan	Retrospective	97	NR	ICDSC	20.6	NLR > 3.0, E-PRE-DELIRIC SCORE > 0.08, ACCI > 5.0, postoperative fentanyl dose ≥ 0.38 µg/kg/hr, BMI < 21 kg/m^2^, ASA 3, blood transfusion	8
Taxis et al. (2022) [15]	Germany	Retrospective	225	NR	DSM-V	21.8	Operative time, ACCI, sex, ICU stay period, impaired wound healing, positive history of nicotine and alcohol abuse, microvascular surgery, previous head and neck surgery, flap success, tracheostomy, postoperative nutritional risk screening score	8
Obermeier et al. (2022) [29]	Germany	Retrospective	198	NR	Nu-DESC	32.8	Duration of intubation, gender, fluid intake	8
Kim et al. (2022) [28]	South Korea	Retrospective	197	60.0 ± 13.3	DSM-V	9.1	Age, past neurological history, time to ambulation	9
Kolk et al. (2022) [5]	Germany	Retrospective	100	65	DSM-V	18.0	Age, diabetes mellitus, preoperative TSH, type of surgery	9

*Abbreviations*: *CAM* Confusion Assessment Method, *DSM-V* Diagnostic and Statistical Manual of Mental Disorders, Fifth Edition, *DSM-IV* Diagnostic and Statistical Manual of Mental Disorders, Fourth Edition, *ICDSC* Intensive Care Delirium Screening Checklist, *Nu-DESC* Nursing Delirium Screening Scale, *NR* Not reported, *NRS* Numeric rating scale of pain, *ASA* American Society of Anesthesiologists, *ICU* Intensive care unit, *ACCI* Age-adjusted Charlson Comorbidity Index, *CCI* Charlson Comorbidity Index, *COPD* Chronic obstructive pulmonary disease, *TSH* Thyrotropic hormone, *NLR* Neutrophil-to-lymphocyte ratio,* BMI* Body mass index, *E-PRE-DELIRIC* Early Prediction Model for Delirium in an intensive care unit

### Methodological quality evaluation

Data on the quality of the eligible articles based on the NOS is presented in the Table 2. The NOS score of all included studies was no less than 7 points, suggesting that all of these were high-quality.

**Table 2:** The Newcastle-Ottawa Scale (NOS) scoring

Study(year)	Representativeness of the exposed cohort	Selection of the non-exposed cohort	Ascertainment of exposure	Outcome not present at baseline	Control for age	Control for other confounding factors	Assessment of outcome	Enough follow-up duration long	Adequacy of follow-up	Total
Shiiba et al. (2009) [18]	1	1	1	1	1	1	1	1	1	9
Hasegawa et al. (2015) [19]	1	1	1	1	1	1	1	1	1	9
Booka et al. (2016) [20]	1	1	1	1	1	1	1	1	1	9
Choi et al. (2017) [27]	1	1	1	1	1	1	1	1	1	9
Zhang et al. (2019) [24]	1	1	1	1	0	1	1	1	1	8
Wang et al. (2019) [25]	1	1	1	1	0	1	1	1	1	8
Ishibashi-Kanno et al. (2020) [12]	0	1	1	1	1	1	1	1	1	8
Densky et al. (2019) [30]	1	1	1	1	1	1	1	1	1	9
Makiguchi et al. (2020) [21]	1	1	1	1	0	1	1	1	1	8
Kong et al. (2021) [26]	0	1	1	1	0	1	1	1	1	7
Takahashi et al. (2021) [22]	1	1	1	1	0	1	1	1	1	8
Kinoshita et al. (2021) [23]	1	1	1	1	0	1	1	1	1	8
Taxis et al. (2022) [15]	1	1	1	1	0	1	1	1	1	8
Obermeier et al. (2022) [29]	1	1	1	1	0	1	1	1	1	8
Kim et al. (2022) [28]	1	1	1	1	1	1	1	1	1	9
Kolk et al. (2022) [5]	1	1	1	1	1	1	1	1	1	9

### Incidence of POD

**Fig. 2:**
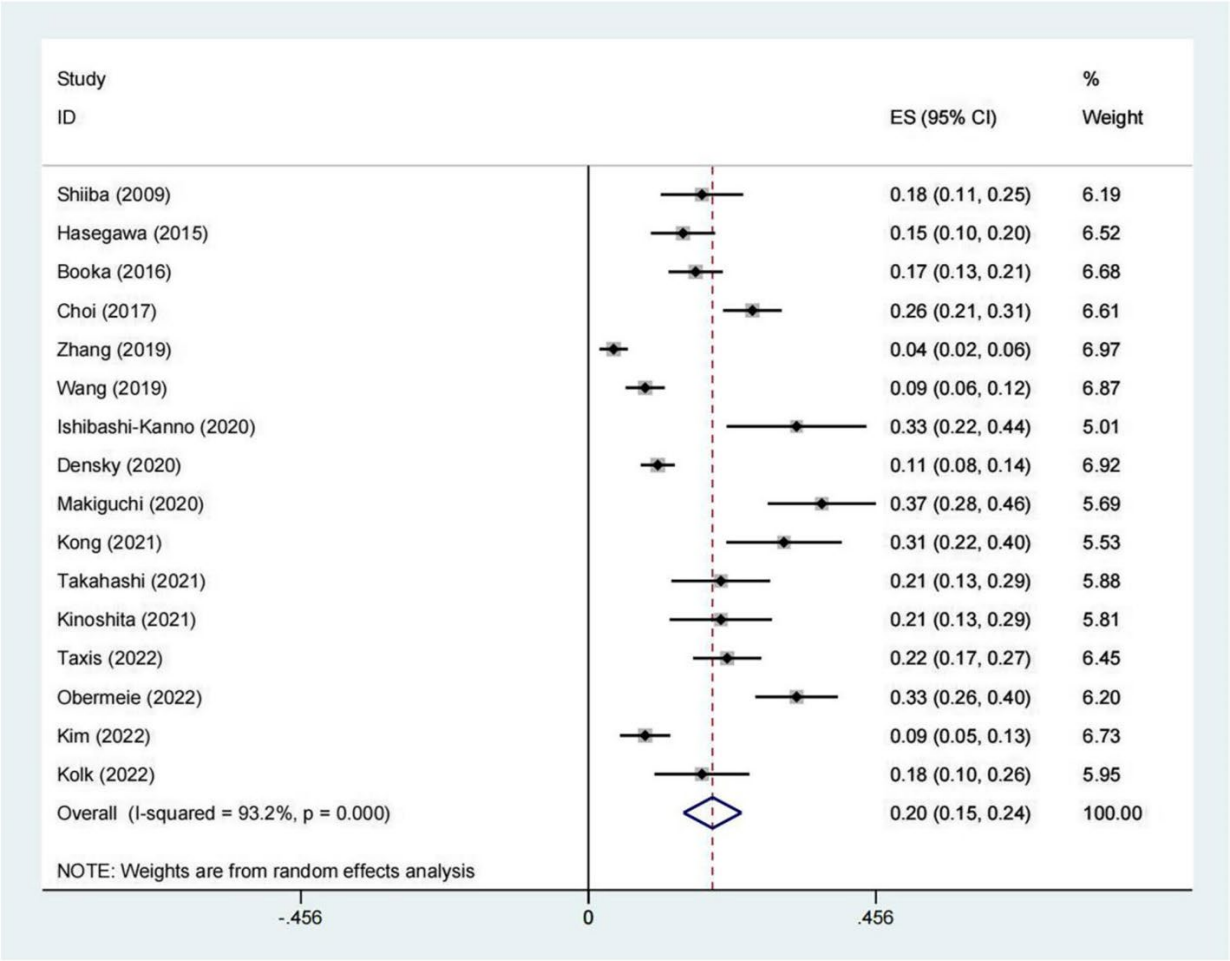
Forest plots of the incidence of postoperative delirium

**Fig. 3:**
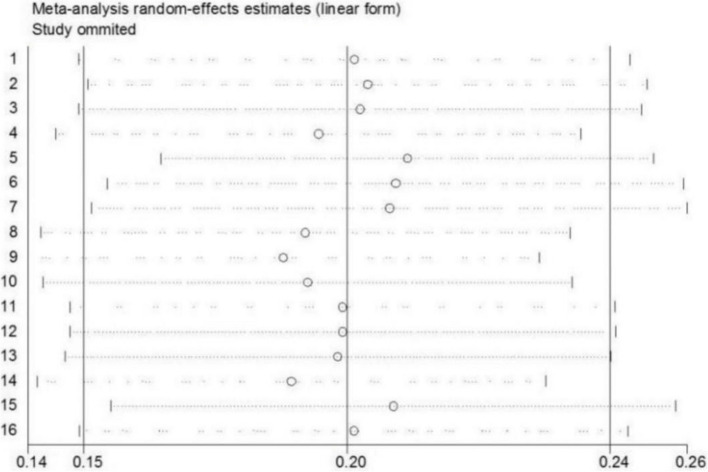
Sensitivity analysis of the incidence of postoperative delirium

**Fig. 4:**
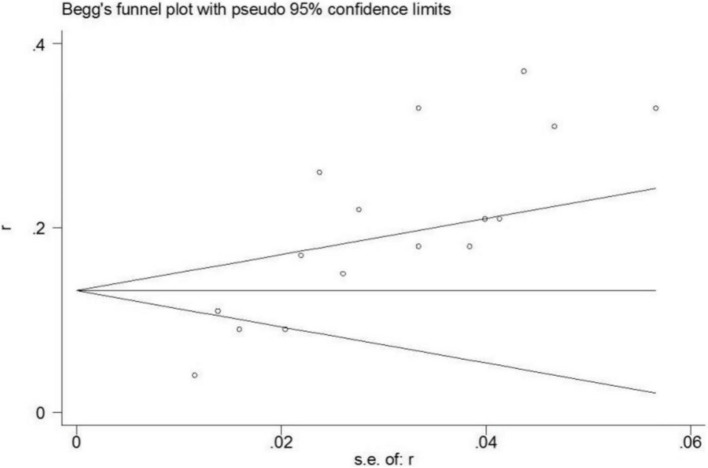
Begg’s funnel plot of the incidence of postoperative delirium

All eligible articles provided the incidence of POD, varying from 4.2 to 36.9% with a pooled incidence of 20% (95% CI 15–24%, I^2^ = 93.2%) (Fig. 2). In addition, subgroup analyses were performed on region, criteria for POD, number of samples, quality of included studies, and study design (Table 3). Furthermore, we performed a sensitivity analysis and the results suggested that none of the included articles had a great influence on the pooled estimates (Fig. 3). Begg’s funnel plot provided significant evidence of publication bias (Fig. 4).

**Table 3:** Subgroup analysis of postoperative delirium after head and neck surgery

Outcomes	Number of studies	ES(95% CI)	I^2^(%)
Pooled results			
Subgroup analyses based on region			
Asia	12	0.18 (0.13–0.22)	93.0
Western countries	4	0.26 (0.19–0.33)	75.4
Subgroup analyses based on criteria for POD			
DSM-IV	8	0.20 (0.13–0.27)	95.0
DSM-V	4	0.16 (0.10–0.22)	81.8
CAM	2	0.20 (0.02–0.41)	95.0
Others	2	0.27 (0.15–0.39)	80.4
Subgroup analyses based on number of sample			
N <200	10	0.23 (0.17–0.29)	87.9
N ≥ 200	6	0.15 (0.08–0.21)	95.1
Subgroup analyses based on quality of included studies			
NOS 9	7	0.19 (0.13–0.24)	84.4
NOS 7–8	9	0.20 (0.15–0.24)	95.1
Subgroup analyses based on study design			
Retrospective	14	0.20 (0.15–0.25)	93.5
Others	2	0.20 (0.02–0.41)	95.0

*Abbreviations*: *CAM* Confusion Assessment Method, *DSM-V* Diagnostic and Statistical Manual of Mental Disorders, Fifth Edition,* DSM-IV* Diagnostic and Statistical Manual of Mental Disorders, Fourth Edition, *POD* Postoperative delirium, *ES* Effect size, *CI* Confidence interval

### Risk factors of POD

Originally, we identified 53 risk factors from included studies based on multivariate analysis. Of these, 8 risk factors were reported in two or more studies, and finally 6 risk factors were considered statistically significant, which are displayed in Table 4. All of these identified factors were divided into 2 categories, including predisposing and precipitating risk factors.

**Table 4:** Meta-analysis of risk factors for postoperative delirium after head and neck cancer surgery

Risk factors	Number of studies	Pooled OR	Lower 95% CI	Upper 95% CI	*P-*value	I^2^ (%)	Statistical method
Age	5	1.05	1.03	1.07	< 0.001	18	Fixed
Age > 75 years	2	6.52	3.07	13.87	< 0.001	0	Fixed
Male gender	5	2.29	1.06	4.97	0.04	55	Random
ASA states	3	2.19	1.44	3.33	< 0.001	31	Fixed
ICU stay	2	2.08	0.46	9.46	0.34	93	Random
Operative time	3	1.00	1.00	1.01	0.003	0	Fixed
History of smoking	2	2.74	1.13	6.65	0.03	0	Fixed
Diabetes mellitus	3	2.73	1.24	6.01	0.01	0	Fixed

*Abbreviations*:* ASA* American Society of Anesthesiologists, *CI* Confidence interval, *ICU* Intensive care unit, *OR* Odds ratio

### Predisposing risk factors

#### Age

**Fig. 5:**
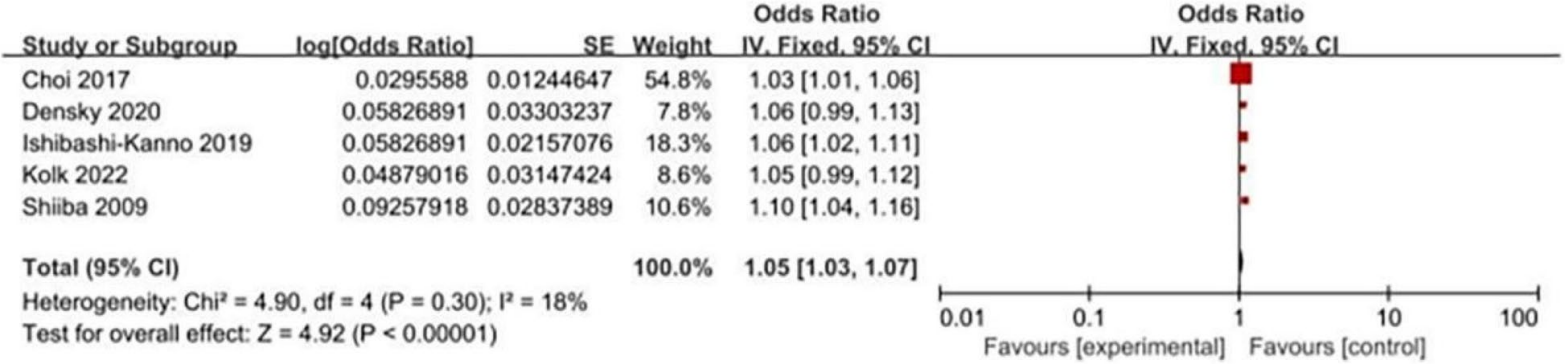
Forest plot for increased age

**Fig. 6:**
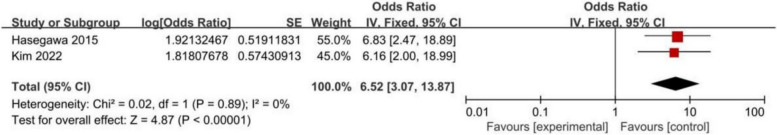
Forest plot for age > 75 years

A total of 5 articles showed that older age was a potential risk factor for POD. The meta-analysis of these articles suggested that older age was a significant predictor for POD (OR: 1.05, 95% CI: 1.03–1.07, *P* < 0.001, I^2^ = 18%, Table 4; Fig. 5). Moreover, age > 75 years was deemed as a risk factor for POD in 2 studies, and the results of this meta-analysis suggested that patients older than 75 years were more prone to experience POD (OR: 6.52, 95% CI: 3.07–13.87, *P* < 0.001, I^2^ = 0%, Table 4; Fig. 6).

### Male sex

**Fig. 7:**
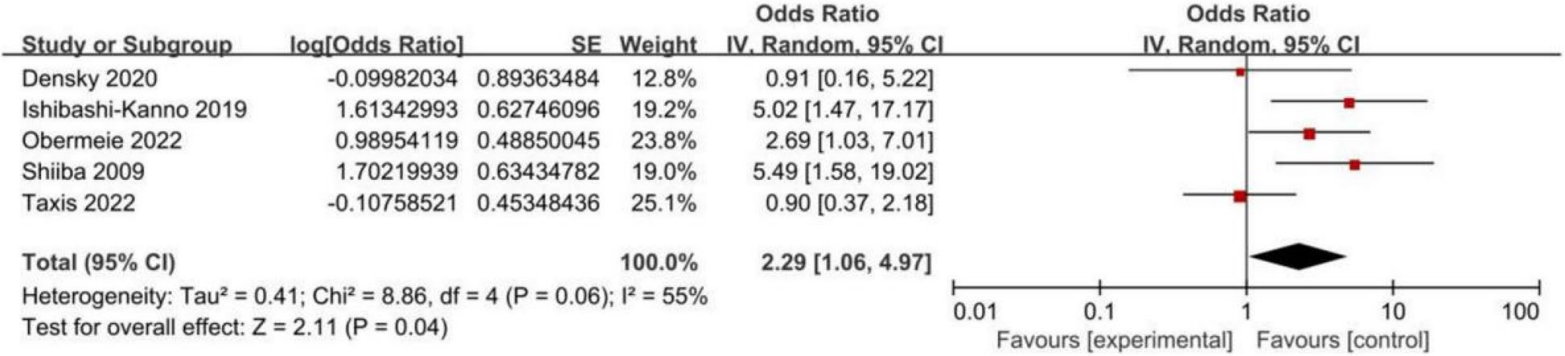
Forest plot for male sex

Five papers reported that male sex was a potential risk factor for POD. The meta-analysis results indicated that male gender was an independent risk factor for POD (OR: 2.29, 95% CI: 1.06-4.97, *P* = 0.04), with mild heterogeneity (I^2^= 55%, *P* = 0.06, Table 4, Fig. 7). Furthermore, the potential sources of heterogeneity were sought through sensitivity analysis, and the results indicated that no single study significantly changed the pooled result.

### American society of anesthesiologists (ASA) physical states

**Fig. 8:**
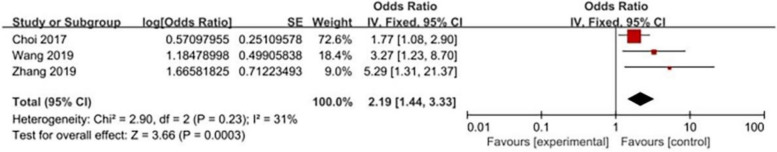
Forest plot for American Society of Anesthesiologists level

Three studies reported the association between ASA physical status and POD, and the meta-analysis indicated that a 2.19-fold increased risk of POD in patients with higher ASA grades (OR: 2.19, 95% CI: 1.44–3.33, *P* < 0.001, I2 = 31%, Table 4; Fig. 8).

### Diabetes mellitus

**Fig. 9:**
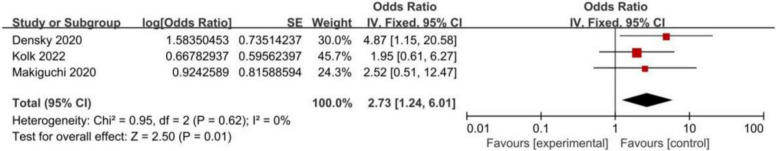
Forest plot for diabetes mellitus

Diabetes mellitus was recognized as a risk factor for POD in 3 papers. In our meta-analysis, patients with diabetes mellitus had a 2.73-fold increased risk of POD compared to patients without diabetes mellitus (OR: 2.73, 95% CI: 1.24–6.01, *P* = 0.01, I2 = 0%, Table 4; Fig. 9).

### History of smoking

**Fig. 10:**
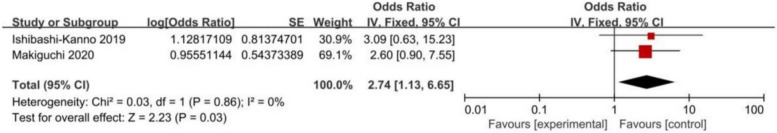
Forest plot for the history of smoking

Two articles reported that the impact of the smoking history on risk of POD. Our results indicated that the pooled OR was 2.74 (95% CI: 1.13–6.65, *P* = 0.03) with no heterogeneity (I2 = 0%, *P* = 0.86, Table 4; Fig. 10).

### Precipitating risk factors

**Fig. 11:**
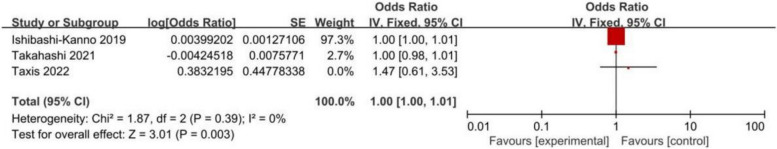
Forest plot for operative time

**Fig. 12:**
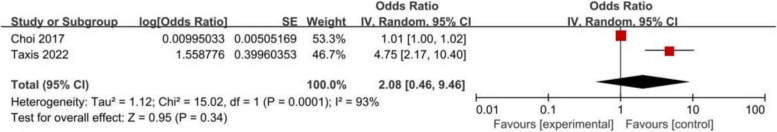
Forest plot for length of intensive care unit stay

In this category, only 2 risk factors were analyzed by meta-analysis, including operative time and ICU stay time. The meta-analysis of 2 risk factors showed that surgery time (OR: 1.00, 95% CI: 1.00-1.01, *P* = 0.003, I2 = 0%, Table 4; Fig. 11) and ICU stay time (OR: 2.08, 95% CI: 0.46–9.46, *P* = 0.34, I2 = 93%, Table 4; Fig. 12) were not significant risk factors for POD.

## Discussion

POD is a common postoperative complication among head and neck cancer surgery patients and is related to notable morbidity and mortality. In light of these adverse prognoses, it is imperative to identify related predictive factors of POD and take appropriate preventive measures to prevent POD in patients undergoing surgery for head and neck cancer. Thus, we conducted this meta-analysis and found several significant predictive factors of POD after head and neck cancer surgery including increased age, age > 75 years, male sex, higher ASA level, diabetes mellitus and history of smoking.

Our study was not the first systematic review and meta-analysis exploring the risk factors for POD after head and neck cancer surgery. Compared with a previous meta-analysis [6], our systematic review and meta-analysis included articles that were published within the latest 5 years and that summarized the incidence of POD after head and neck cancer surgery. The prevalence of POD varies significantly among surgical populations; for instance, the incidence of POD is reportedly 5.45 to 28.57% after urological surgery, 10.09 to 51.28% after hip fracture surgery, and 4.1 to 54.9% after cardiac surgery [13, 14, 32]. In our study, the incidence of POD ranged from 4.2 to 36.9% with a pooled incidence of 20% and high heterogeneity (I2 = 93.2%, *P* < 0.001), which was consistent with a previous meta-analysis [6]. The heterogeneity of POD incidence may be a consequence of the sample size, the region of surgery, or the POD diagnostic criteria [33]. In addition, the incidence of POD in Western countries was significantly higher than that in Asia, possibly due to ethnic differences.

It is widely accepted that advanced age is an important predisposing predictor for POD [34]. In this meta-analysis, older age was associated with a relatively low risk of POD after head and neck cancer surgery. However, for patients over 75 years old, the risk of POD was 6.52 times higher than that in patients younger than 75 years old, which was in line with a previous meta-analysis [35]. The results may be explained by the presence of more comorbidities such as depression, preexisting cerebrovascular disease, insomnia, and frailty in elderly patients [36]. An alternative explanation might be age-related inflammatory response changes, which might play a role in the pathophysiology of POD [37]. In addition, the effect of sex differences on POD risk remains controversial. A previous meta-analysis suggested that female sex was a predictor for POD in patients undergoing spinal surgery [38]. Conversely, our results indicated that male sex was significantly associated with POD development in head and neck cancer surgery patients. A possible explanation was that males were more likely to develop obstructive sleep apnoea and alcohol dependence, which have been confirmed as significant risk factors for POD [39].

ASA classification is a well-known grading system for evaluating patients’ tolerance to anesthesia and their physical status before surgery. In line with our recent study [40], our results indicated that an ASA level increase was a strong predictor for POD. Therefore, those who have a higher ASA level should be of great concern to clinicians. In our study, we also investigated whether diabetes mellitus was associated with POD after head and neck cancer surgery. Diabetes mellitus is a well-established risk factor for the development of dementia [41]. Additionally, the study by Liu and colleagues demonstrated that the increased risk of POD after hip fracture surgery was explained by diabetes mellitus [42]. Our results also revealed that head and neck cancer surgical patients with diabetes mellitus were more susceptible to POD. Diabetes mellitus could weaken insulin signaling pathways in the regulation of the functions of neurons and glial cells [43]. In addition, diabetes mellitus is characterized by hyperglycemia, oxidative stress, and chronic inflammation that can lead to blood-brain barrier impairment [44]. Thus, it is not surprising that patients with diabetes mellitus had a higher risk for POD after head and neck cancer surgery.

Several studies have demonstrated that a history of smoking was associated with surgical complications, including postoperative pneumonia and wound infection [45]. However, the correlation between a history of smoking and perioperative neurocognitive disorders remains obscure to date. A previous study showed that a preoperative smoking history could decrease the incidence of early postoperative cognitive dysfunction by stimulating the cholinergic anti-inflammatory pathway [31]. Interestingly, our results suggested that a history of smoking was considered as a significant predictor for POD after head and neck cancer surgery. Similar to our current study, Zhou et al. also found that smoking was positively related to POD in patients undergoing non-cardiac and non-obstetric surgery [46], which was associated with the impaired cholinergic function due to nicotine withdrawal resulting from sudden cessation of smoking [47].

This meta-analysis did not conclude that operative time and intensive care unit duration were predictive factors of POD after head and neck cancer surgery, which might be related to the small number of studies included. However, according to the Consensus-based Guideline on POD of the European Society of Anesthesiology, operative time and ICU stay time should be considered as risk factors for POD after surgery [48]. Thus, the connection between the 2 risk factors and POD needs to be validated in future large-sample prospective multicentre cohort studies.

### Limitations

There were some limitations in our study. First, only articles published in English were included in the current study, resulting in unavoidable selection bias. Second, we identified several risk factors for POD after head and neck cancer surgery, however, the association between these risk factors and POD in other surgical populations still need to be explored further. Third, some significant risk factors were identified in a few studies with small sample sizes, which should be interpreted with caution.

## Conclusions

To sum up, our meta-analysis indicated that POD was common after head and neck cancer surgery. Based on the multivariate analysis, some significant predictors were identified, including increased age, age > 75 years, male sex, higher ASA grade, diabetes mellitus, and history of smoking, which might play a critical role in optimizing clinical management of POD.

### Supplementary Information

**Additional file 1.** 

## Data Availability

All data generated or analyzed during this study are included in this published article and its supplementary information files.

## Abbreviations

POD: Postoperative delirium
NOS: Newcastle-Ottawa scale
OR: Odds ratio
CIs: Confidence intervals
Nu-DESC: Nursing Delirium Screening Scale
ICDSC: Intensive Care Delirium Screening Checklist
CAM: Confusion Assessment Method
DSM: Diagnostic and Statistical Manual of Mental Disorders
ASA: American Society of Anesthesiologists
