# Supplementary material for: Incidence and risk factors for postoperative delirium after head and neck cancer surgery: an updated meta-analysis
Source: BMC Neurol. 2023 Oct 17;23:371. doi: 10.1186/s12883-023-03418-w (PMC10580509; doi:10.1186/s12883-023-03418-w)
Supplement: Supplementary file 1 — Additional file 1. [file 12883_2023_3418_MOESM1_ESM.docx]

**Each database retrieval strategy**

**PUBMED**

| **Search** | **Query** | **Results** |
| --- | --- | --- |
| # 1 | delirium[MeSH Terms] OR postoperative delirium[Title/Abstract] OR acute confusional syndrome[Title/Abstract] OR subacute deliriums[Title/Abstract] OR transient mental disorder[Title/Abstract] OR mixed origin delirium[Title/Abstract] OR confusion*[Title/Abstract] | 51550 |
| # 2 | head and neck[Title/Abstract] OR oral[Title/Abstract] OR mouth[Title/Abstract] OR tongue[Title/Abstract] OR laryngitis*[Title/Abstract] OR pharynx*[Title/Abstract] OR nasopharyngeal*[Title/Abstract] OR hypopharyn*[Title/Abstract] OR oropharynx*[Title/Abstract] | 926256 |
| # 3 | neoplasms[MeSH Terms] OR cancer[Title/Abstract] OR tumor[Title/Abstract] OR malignancy[Title/Abstract] OR carcinoma[Title/Abstract] | 51550 |
| # 4 | #2 AND #3 | 237024 |
| # 5 | risk factors[MeSH Terms] OR risk[Title/Abstract] OR factor*[Title/Abstract] OR predict*[Title/Abstract] | 7085486 |
| # 6 | #1 AND #4 AND #5 | 122 |

EMBASE

| **Search** | **Query** | **Results** |
| --- | --- | --- |
| # 1 | 'delirium'/exp OR 'postoperative delirium':ab,ti OR 'acute confusional syndrome':ab,ti OR 'subacute deliriums':ab,ti OR 'transient mental disorder':ab,ti OR 'mixed origin delirium':ab,ti OR 'confusion*':ab,ti | 101354 |
| # 2 | 'head and neck':ab,ti OR 'oral':ab,ti OR 'mouth':ab,ti OR 'tongue':ab,ti OR 'laryngitis*':ab,ti OR 'pharynx*':ab,ti OR 'nasopharyngeal*':ab,ti OR 'hypopharyn*':ab,ti OR 'oropharynx*':ab,ti | 1257864 |
| # 3 | 'neoplasms'/exp OR 'cancer':ab,ti OR 'tumor':ab,ti OR 'malignancy':ab,ti OR 'carcinoma':ab,ti | 4541928 |
| # 4 | #2 AND #3 | 273896 |
| # 5 | 'risk factors'/exp OR 'risk':ab,ti OR 'factor*':ab,ti OR 'predict*':ab,ti | 9285161 |
| # 6 | #1 AND #4 AND #5 | 354 |

COCHRANE LIBRARY

| **Search** | **Query** | **Results** |
| --- | --- | --- |
| # 1 | MeSH descriptor: [Delirium] explode all trees | 1057 |
| # 2 | (postoperative delirium):ti,ab,kw OR (acute confusional syndrome):ti,ab,kw OR (subacute deliriums):ti,ab,kw OR (transient mental disorder):ti,ab,kw OR (mixed origin delirium):ti,ab,kw OR (confusion*):ti,ab,kw | 5471 |
| # 3 | #1 OR #2 | 5849 |
| # 4 | (head and neck):ti,ab,kw OR (oral):ti,ab,kw OR (mouth):ti,ab,kw OR (tongue):ti,ab,kw OR (laryngitis*):ti,ab,kw OR (pharynx*):ti,ab,kw OR (nasopharyngeal*):ti,ab,kw OR (hypopharyn*):ti,ab,kw OR (oropharynx*):ti,ab,kw | 220641 |
| # 5 | MeSH descriptor: [Neoplasms] explode all trees | 90677 |
| # 6 | (cancer):ti,ab,kw OR (tumor):ti,ab,kw OR (malignancy):ti,ab,kw OR (carcinoma):ti,ab,kw | 230815 |
| # 7 | #5 OR #6 | 251982 |
| # 8 | #4 AND #7 | 35734 |
| # 9 | MeSH descriptor: [Risk Factors] explode all trees | 26424 |
| # 10 | (risk):ti,ab,kw OR (factor*):ti,ab,kw OR (predict*):ti,ab,kw | 513234 |
| # 11 | #9 OR #10 | 513234 |
| # 12 | #3 AND #8 AND #11 | 66 |
